# Molecular Muscle Experiment: Hardware and Operational Lessons for Future Astrobiology Space Experiments

**DOI:** 10.1089/ast.2019.2181

**Published:** 2020-08-06

**Authors:** Amelia K. Pollard, Christopher J. Gaffney, Colleen S. Deane, Michele Balsamo, Michael Cooke, Rebecca A. Ellwood, Jennifer E. Hewitt, Beata E. Mierzwa, Alessandro Mariani, Siva A. Vanapalli, Timothy Etheridge, Nathaniel J. Szewczyk

**Affiliations:** ^1^ MRC Versus Arthritis Centre for Musculoskeletal Ageing Research and NIHR Nottingham BRC, University of Nottingham, Medical School Royal Derby Hospital, Derby, United Kingdom.; ^2^ Sport and Health Sciences, University of Exeter, Exeter, United Kingdom.; ^3^ Lancaster Medical School, Furness College, Lancaster University, Lancaster, United Kingdom.; ^4^ Kayser Italia, Livorno, Italy.; ^5^ Department of Chemical Engineering, Texas Tech University, Lubbock, Texas.; ^6^ Department of Cellular and Molecular Medicine, Ludwig Institute for Cancer Research, University of California San Diego, La Jolla, California.

**Keywords:** Spaceflight, Space biology, Astrobiology, *C. elegans—*

## Abstract

Biology experiments in space seek to increase our understanding of what happens to life beyond Earth and how we can safely send life beyond Earth. Spaceflight is associated with many (mal)adaptations in physiology, including decline in musculoskeletal, cardiovascular, vestibular, and immune systems. Biological experiments in space are inherently challenging to implement. Development of hardware and validation of experimental conditions are critical to ensure the collection of high-quality data. The model organism ***Caenorhabditis elegans*** has been studied in space for more than 20 years to better understand spaceflight-induced (patho)physiology, particularly spaceflight-induced muscle decline. These experiments have used a variety of hardware configurations. Despite this, hardware used in the past was not available for our most recent experiment, the Molecular Muscle Experiment (MME). Therefore, we had to design and validate flight hardware for MME. MME provides a contemporary example of many of the challenges faced by researchers conducting ***C. elegans*** experiments onboard the International Space Station. Here, we describe the hardware selection and validation, in addition to the ground-based experiment scientific validation testing. These experiences and operational solutions allow others to replicate and/or improve our experimental design on future missions.

## 1. Introduction

Space is an extreme environment in which humans have been routinely living and working for more than 20 years, and ambitions remain to live and work further beyond Earth. Spaceflight is associated with deconditioning of multiple bodily systems, including the musculoskeletal, cardiovascular, vestibular, and immune systems (Demontis *et al.*, 2017; Strollo *et al.*, 2018). Physiological decline occurs despite participation in exercise countermeasures, which are a potent stimulus to promote musculoskeletal and cardiovascular adaptation (Trappe *et al.*, 2009) and optimal immune function (Simpson *et al.*, 2015) on Earth. While vestibular adaptations take place in the first days after transition to the microgravity environment, decline in musculoskeletal, cardiovascular, and immune systems can worsen with missions of greater duration (Fitts *et al.*, 2010; Strollo *et al.*, 2018), potentially limiting our capacity for interplanetary missions such as to Mars (Gaffney *et al.*, 2017).

These challenges have motivated research around strategies to prevent this (mal)adaptation in space. As the precise molecular mechanisms of spaceflight-induced physiological deconditioning have not been identified, this limits our ability to design molecularly rationalized countermeasures.

Biological experiments in space can, however, be methodologically challenging. Although the United States has designated the US segment of the International Space Station (ISS) as a National Laboratory, experiments are not routinely carried out by using standardized equipment or methods that are in common use in other laboratories on Earth. Size, power, mass, late access for experiment integration onto the rocket, and ability to oversee the experiment remain challenges, despite more than 40 years of conducting biology experiments in space. Thus, the development of hardware and the validation of experimental conditions remain critical in the acquisition of quality data that can ultimately promote understanding of biological changes in space that should underpin rationale countermeasures to physiological changes in space.

Our current experiment, the Molecular Muscle Experiment (MME), uses the model organism, *Caenorhabditis elegans*, to explore the molecular mechanisms of spaceflight-induced muscle atrophy. *C. elegans* is an established model organism for studies on Earth (Kaletta and Hengartner, 2006; Corsi *et al.*, 2015; Shen *et al.*, 2018) and in space (Szewczyk *et al.*, 2008; Higashitani *et al.*, 2009; Honda *et al.*, 2012, 2014; Higashibata *et al.*, 2016).

In the context of very limited access to space, the short life span and microscopic size of *C. elegans* enable data to be collected across the entire life span (Gaffney *et al.*, 2018a) in high *n* numbers and replicates to generate robust data rapidly. The operational requirements of MME include many that apply to biological payloads across species (*e.g.*, late access, temperature-controlled upload/download, and time critical astronaut interventions). MME is therefore a generalizable example of many of the challenges in conducting contemporary astrobiology research onboard the ISS.

*C. elegans* have been studied in space for more than 20 years by using a variety of spaceflight hardware configurations (Ishioka and Higashibata, 2019). With the exception of STS-107 (Szewczyk *et al.*, 2005), all past flights involved in the study of *C. elegans* have used nonstandard laboratory hardware. It is therefore not surprising that *C. elegans* spaceflight experiments can fail due to lack of sufficient oxygen to support the worms (Warren *et al.*, 2013), a problem that can be dealt with through preflight ground testing. Here, we describe the hardware selection and validation in addition to the ground-based experiment sequence validation testing for MME.

The first key challenge was overcoming the difficulty of growing worms inside plastic bags. The second key challenge was working out timings to allow upload to the ISS using passive temperature control. These studies should allow other researchers to replicate and/or improve our experimental design on future missions.

## 2. Materials and Methods

### 2.1. Nematode and bacteria preparation

The *C. elegans* strain PD55 (*ccIs55* (*unc-54::lacZ*) V) was used for development and testing phases of this study. Animals were cultured at 20°C by using standard methods on nematode growth media plates. To obtain L1 animals, age synchronizations were carried out by gravity settling as described previously (Gaffney *et al.*, 2014). During the hardware development phases, *Escherichia coli* OP50 was grown overnight in LB-broth, centrifuged, and resuspended in S-Basal; 500 or 5000 L1 were used in experiments with 3–10 mL of OP50.

During the hardware development phases and all experimentation unless stipulated otherwise, stocks were prepared by adding 5000 L1 larvae to 6 mL of freeze-dried OP50 (LabTie; StartLife, Netherlands) in S-Basal. Freeze-dried OP50 was made by using LabTie's protocol of 1 vial per 250 mL S-Basal. To streamline procedures for launch preparation, worms to be flown (PD55 (*ccIs55* (*unc-54::lacZ*) V), CF1038 (*daf-16* (*mu86*) I), CF1139 (*daf-16* (*mu86*) I; *muIs61* (*daf-16::GFP*)?), PJ1145 (*ccIs55* (*unc-54::lacZ*) V; *njEx38* (*unc-54p::daf-2(+)* + *goa-1p::GFP* + *rol-6* (*su1006*))), DM2 (*dim-1* (*ra102*) X)) were cultured in freeze-dried OP50 in S-Basal in 75 cm^2^ cell culture flasks. Animals were cultured for a minimum of 2 weeks following transfer from plates before synchronization.

During final preflight testing of the launch procedures, age synchronizations were carried out from liquid culture stocks rather than from plates to obtain 5000 L1 larvae the day before loading into bags. These cultures were left overnight in 6-well tissue culture dishes in a final volume of 500 μL freeze-dried OP50 in S-Basal at 20°C. The next day, 6 mL freeze-dried OP50 was pipetted into each well before loading into flight culture bags.

### 2.2. Validation of culture bags

The experimental design for launch involved growing worms inside gas-permeable plastic bags onboard the ISS. Therefore, we tested the biocompatibility of three different bag materials: (i) polyethylene (PE) bags (1.5 inch, 250 gauge, Lay Flat Polythene Tubing; Kite Packaging Ltd., United Kingdom), (ii) Japanese Aerospace Exploration Agency (JAXA) PE bags (Nipro, Japan), and (iii) fluorinated ethylene propylene (FEP) bags (Saint-Gobain Performance Plastics; TOSS GmbH, Germany). The PE bags and FEP bags are low friction and allow good gas exchange of oxygen and carbon dioxide, although gas exchange is reported to be superior in FEP to PE in bags of equal thickness (NB. all bags have previously supported *C. elegans* cultures in spaceflight experiments onboard the ISS).

The JAXA bags are a proprietary PE blend that is no longer commercially available. To test the bags, 5000 L1 larvae were placed in the bag with OP50 before bags were heat sealed (Pacseal^+^ impulse heat sealer; Rajapack, United Kingdom), and then, the worms were cultured for 7 days at 20°C. After 7 days, the total worm population in each bag was counted. Three biological replicates were carried out per condition. A one-way analysis of variance (ANOVA) was carried out in GraphPad Prism to compare the populations of worms in the three bags.

### 2.3. Oxygen consumption rates

To investigate whether diet influenced changes in oxygen consumption rates (OCR), measurements were carried out with the Seahorse XFe24 Analyzer (Agilent, Santa Clara, CA), as previously described (Hewitt *et al.*, 2018; Koopman *et al.,* 2016). Worms were cultured in either chemically defined media [*C. elegans* maintenance media, CeMM (Szewczyk *et al.*, 2003), purchased from Cell Guidance Systems custom media service, United Kingdom], or freeze-dried OP50 in S-Basal. Adult animals were washed twice in incubation media [M9 buffer: 22.04 mM KH_2_PO_4_, 42.27 mM Na_2_HPO_4_, 85.56 mM NaCl (final concentrations, sterilized)] and placed in 200 μL M9-filled wells of standard Seahorse plates (20 worms/well) in 5 replicates per condition. Stable OCR measurements were taken by performing five measurement cycles for basal OCR, nine cycles for maximal OCR following the addition of carbonyl cyanide p-trifluoromethoxyphenylhydrazone (FCCP) (10 μM final well concentration), and five cycles for nonmitochondrial OCR following the addition of sodium azide (40 nM final well concentration).

OCR measurements were normalized to the number of worms per well. To obtain stable OCR measurements, the final three, seven, and two measurement cycles were used for the statistical analysis of basal, maximal, and nonmitochondrial OCR, respectively, as previously described (Hewitt *et al.*, 2018). Differences in OCR were detected with a one-way ANOVA with Tukey's multiple comparison test using GraphPad Prism 8 (La Jolla, CA). Statistical significance was set at *p* < 0.05.

### 2.4. Culture volume optimization

Age-synchronized L1 larvae were placed into experimental bags containing freeze-dried OP50 in S-Basal (5000 L1 worms per bag). The amount of OP50 in the bag was optimized where 3, 4, 5, 6, 7, 8, 9, and 10 mL volumes were tested. Three biological replicates were carried out per condition.

### 2.5. Hardware development and validation

**FIG. 1.**
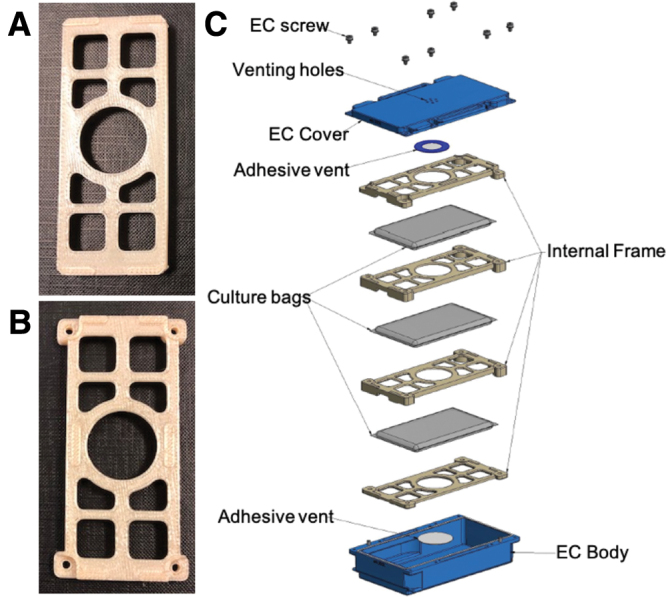
Hardware for the MME. Images of the hardware used for the experiment include **(A)** the first cage prototype and **(B)** the second cage prototype developed by Kayser Italia. **(C)** The assembly of experimental bags and cages within the EC. EC, experiment cassette; MME, Molecular Muscle Experiment. Color image is available online.

For flight, the experimental bags were to be placed into European Space Agency (ESA) experiment cassettes (ECs). As *C. elegans* require oxygen to develop, gas exchange between the inside of the EC and outside of the EC is required. ECs that allowed gas exchange via a gas-permeable Gortex membrane have previously been used with *C. elegans* onboard the ISS (Szewczyk *et al.*, 2008); unfortunately, these were no longer available for use with MME. Therefore, we had to design (Fig. 1) and validate new ECs (Kayser Italia, Livorno, Italy).

To validate how well worms would grow inside the ECs, three bags each loaded with 5000 L1 worms and 6 mL freeze-dried OP50 in S-Basal were cultured inside the EC for 1 week at 20°C. Once complete, the population of viable worms in the bags were counted under a dissecting microscope (Nikon SMZ645; Nikon, Japan) and compared against control bags that were otherwise identical except were located outside the EC.

Two different cage inserts for the ECs were designed and tested to maintain separation between the bags during the turbulent upload phase of launch, to maintain a surface area for gas exchange in flight, and to prevent the bags from freezing together at the end of the experiment. To test which cage design was best, worms were grown in bags alone, in cage design 1, and in cage design 2. Upon selection of cage design 2, further testing was carried out by growing worms in bags alone, in cage 2 alone, and in cage 2 inside an EC. Three biological replicates were carried out per condition.

### 2.6. Validation of experimental timings

As MME was to be uploaded in an active state (*e.g.*, worms were not placed in stasis), and as launches to the ISS are often associated with delays due to inclement weather or technical problems, we needed to validate our experimental design to confirm that it could withstand launch delays. To test this, we obtained upload times from handover to installation on the ISS from NASA cold stowage for previous SpaceX launches (Table 1), as well as data for the performance of 10°C and 12°C ICE Bricks passive upload cold stowage phase change material over upload to ISS time frames.

**Table 1.** Duration from Launch: 26 H Turnover to NASA Cold Stowage Until Installation on the International Space Station

Mission	Handover to installation, h
SpX-4	130
SpX-5	97
SpX-6	137
SpX-8	91
SpX-9	105
SpX-10	152
SpX-11	138
SpX-12	90
SpX-13	94
SpX-14	94

Using these data, we loaded 5000 L1 larvae into 6 mL of freeze-dried OP50 in S-Basal into the bags inside the full EC setup (including the cage insert) and tested various upload scenarios. Following upload scenario testing, we ran the full experiment simulating a scheduled launch, a 24 h delay, and a 48 h delay. Following these simulations, the total worm populations were counted and compared with the nominal (on-time) launch scenario. A nominal launch scenario is associated with the generation of >45,000 worms per bag, which is the requirement for postflight analysis. To determine whether our experimental design could withstand a 24 or 48 h delay, we set a matched population threshold of >45,000 worms per bag at the end of the experiment.

## 3. Results

### 3.1. Evaluation and selection of culture bags

Several previous spaceflight experiments have grown worms in liquid culture inside plastic experimental bags (Selch *et al.*, 2008; Szewczyk *et al.*, 2008; Higashitani *et al.*, 2009); in some cases, worms were grown with an OP50 diet, in others with a CeMM diet. While it is not usual to grow *C. elegans* inside plastic bags on Earth, containment of liquid is a safety requirement onboard the ISS. We therefore sought to establish a plastic culture bag that would allow worm growth for MME.

**FIG. 2.**
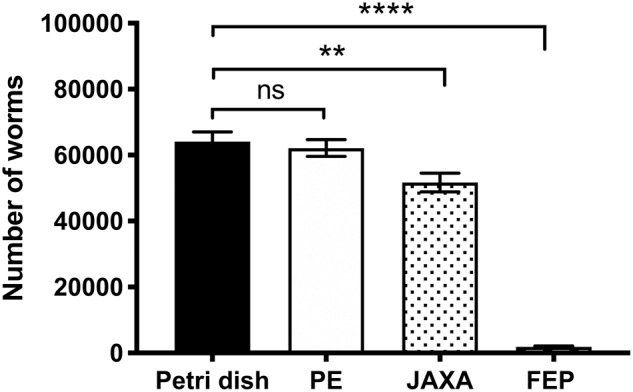
Growth of *Caenorhabditis elegans* in different plastic culture bags. Five thousand L1 larvae were loaded in standard Petri dishes, PE bags, JAXA bags, or FEP bags and cultured for 1 week at 20°C (all conditions in triplicate). Growth in PE bags was not significantly different from Petri dish controls, whereas both JAXA (**p < 0.01) and FEP (****p < 0.0001) have reduced growth compared with controls. Both PE and JAXA produce viable numbers of worms for the analysis requirements, whereas FEP do not. FEP, fluorinated ethylene propylene; JAXA, Japanese Aerospace Exploration Agency; PE, polyethylene.

We evaluated three culture bags based on past use in *C. elegans* flight experiments. Unsurprisingly, the PE bags we selected to replicate the bags used on ICE-FIRST (Szewczyk *et al.*, 2008) and the JAXA bags we selected to replicate the bags used on CERISE (Higashitani *et al.*, 2009) performed well at supporting worm growth (Fig. 2). However, the FEP bags we selected to replicate bags tested on Earth (Szewczyk *et al.*, 2003) for flight use failed to support growth, with more than half of the worms actually dying in the bags (Fig. 2).

### 3.2. Diet induces changes in OCR

Given that past ground tests with FEP bags (Szewczyk *et al.*, 2003) and a past flight with culture chambers utilizing FEP for gas exchange (Oczypok *et al.*, 2012) successfully supported *C. elegans* growth, we wanted to understand why FEP bags did not work in our ground testing (Fig. 2). Since both past reports of use of FEP involved growing worms in CeMM rather than the OP50 diet used in MME, we were curious if the failure to grow was due to the food source. Worms grown in CeMM take longer to develop, have longer life expectancies, exhibit altered gene expression, and exhibit the characteristics of dietary restriction (Szewczyk *et al.*, 2006; Zhang *et al.*, 2015).

**FIG. 3.**
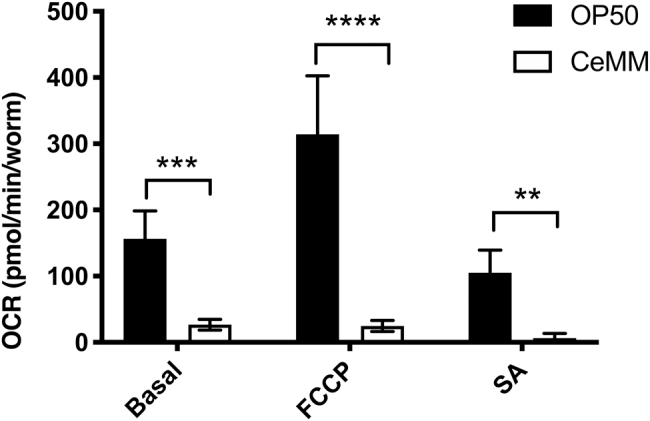
*C. elegans* grown on a bacterial diet display increased oxygen consumption compared with a chemically defined diet. Twenty adult worms grown on bacterial diet (OP50) or chemically defined diet (CeMM) were analyzed in a Seahorse XF Analyzer (*n* = 5 replicates per condition). In both the basal state (***p < 0.001) and in the maximal state following injection of FCCP (****p < 0.0001), oxygen consumption rates are significantly higher in OP50 compared with CeMM. **p < 0.01. CeMM, *C. elegans* maintenance media; FCCP, carbonyl cyanide p-trifluoromethoxyphenylhydrazone; SA, sodium azide.

Given the physiological and gene expression changes in CeMM-grown worms, we hypothesized that worms grown in CeMM have differences in metabolism compared with those grown in OP50. To examine whether these different diets alter metabolism, we used the Seahorse XFe24 Analyzer (Agilent) to measure oxygen consumption in worms grown chronically in CeMM and OP50. As shown in Fig. 3, worms grown in CeMM have significantly lower basal levels of oxygen consumption. This suggests that OP50-grown worms could have been limited for oxygen inside the FEP bags, whereas the CeMM grown worms were not. The use of CeMM rather than OP50 as a food source may therefore reduce the oxygen exchange requirement and thus increase the viability of worm experiments conducted inside plastic culture bags on the ISS.

### 3.3. Optimizing culture volume in the flight bags

**FIG. 4.**
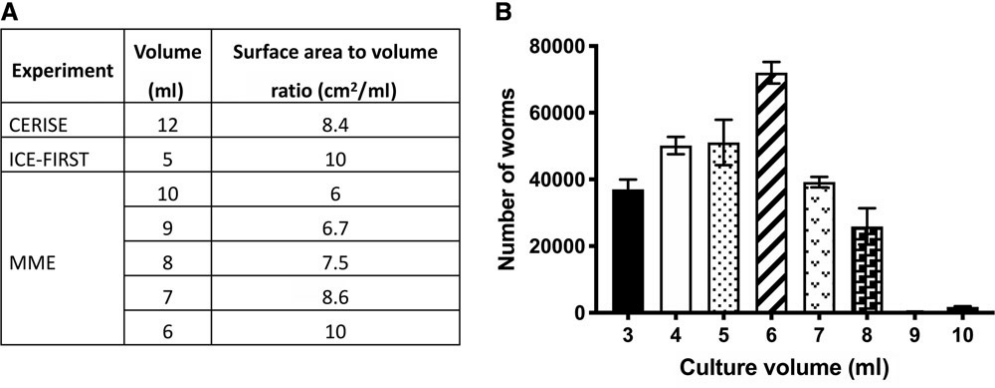
Optimization of culture volume in PE bags. **(A)** Volume of liquid culture and surface area to volume ratio in CERISE, ICE-FIRST, and MME. **(B)** Worm population size after starting with 5000 L1 worms, which were allowed to grow for 7 days at 20°C (*n* = 3 replicates), using different culture volumes in PE. Worm growth is optimal between 5 and 7 mL culture volume with a surface area to volume ratio in the range of 10 cm^2^/mL.

Having determined that gas exchange was a limiting factor for worm growth inside plastic culture bags, we re-evaluated the surface area to volume ratio of our bags versus past flights CERISE (Higashitani *et al.*, 2009) and ICE-FIRST (Szewczyk *et al.*, 2008) as this ratio also influences oxygen availability for the worms within the bags. As shown in Fig. 4, the surface area to volume ratio for CERISE, which used the OP50 diet, was 8.4 cm^2^/mL, whereas the ratio for ICE-FIRST, which used the CeMM diet, was 10 cm^2^/mL. We therefore maintained the size of the bag and altered the volume of worms + OP50 feed solution, testing volumes between 3 and 10 mL. Based on CERISE, which used JAXA bags, we expected a ratio of around 8.4 cm^2^/mL, which equates to a little more than 7 mL of liquid culture in the bag.

To preserve the limited available stocks of JAXA bags and to validate materials readily available for future flights, we used PE bags instead of the JAXA bags for testing. Testing with PE bags (Fig. 4) revealed that the maximum volume in the PE culture bag should be 6–7 mL, which is roughly consistent with the past performance of the JAXA bags during CERISE. Together, these results highlight the importance of considering and testing surface area for gas exchange when designing *C. elegans* spaceflight experiments involving liquid cultures.

### 3.4. Flight hardware validation

**FIG. 5.**
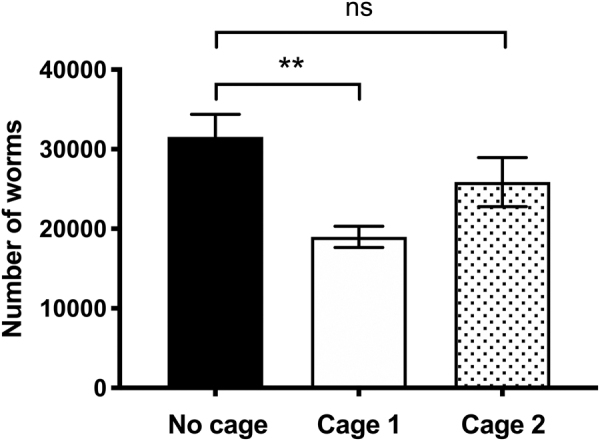
Growth of worms within prototype cages. Five hundred L1 worms were grown in culture bags for 7 days at 20°C, either within or not within a cage design (*n* = 3 replicates). Worm growth was lower in cage 1 compared with no cage (**p < 0.01), whereas there was no significant difference in worm growth between no cage and cage 2.

The culture bags were to be held within ECs to provide an additional level of containment, thus increasing the safety of the crew as well as protecting the cultures themselves from launch and/or in-flight damage. As shown in Fig. 1, cages to contain the bags within the ECs were designed to enforce space between the culture bags during launch, ensure gas exchange, and prevent culture bags from freezing together at the end of the experiment. As shown in Fig. 5, when culture bags were used either outside or inside cage design 2, which had more space between the bags, there was no significant difference in worm growth. In contrast, cage design 1, which had less space between the bags, had significantly less growth than outside the cage. Thus, cage design 2 was adopted for MME.

**FIG. 6.**
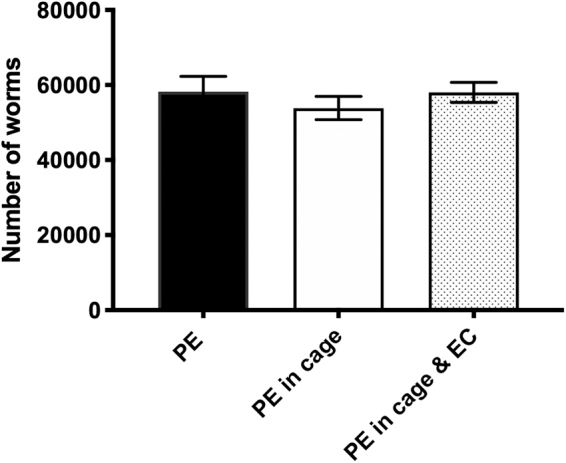
Validating growth of worms within the full experimental design. Five thousand L1 worms were grown in PE culture bags for 7 days at 20°C (*n* = 3 replicates). Three bags were grown without additional hardware, three bags were placed in the cage without the cassette, and three bags were placed in the complete experimental container (cage and cassette, Fig. 1). No significant differences in growth were observed.

Next, growth in the culture bags inside the ECs with or without the cages was evaluated. As shown in Fig. 6, no significant differences in worm growth were noted for worms grown in PE culture bags outside or inside cage design 2, confirming prior testing (Fig. 5). Furthermore, there were no significant differences for worms grown in PE culture bags alone versus inside the combined cage and ECs.

### 3.5. Optimizing culture conditions for upload

To allow for comparison of our anticipated gene expression data with gene expression data from ICE-FIRST (Szewczyk *et al.*, 2008) and CERISE (Higashitani *et al.*, 2009), it was important that we time our cultures to enter adulthood following birth and development in space. As our experiment did not involve an activation step like CERISE, we conducted it as a timed upload like ICE-FIRST. However, for MME, the launch vehicle was SpaceX's Dragon and not Russia's Soyuz, and active temperature control during upload was not preferred from an operations perspective.

Therefore, as SpaceX's Dragon is frequently subjected to launch delays and as phase change materials could be used to maintain temperature during upload, it was important that we test various upload scenarios to establish working timings for upload to the ISS such that the worms were at an appropriate developmental stage, population density, and had not run out of food. As shown in Table 2, we started testing with fixed temperature of a duration calculated to allow P_0_ animals to grow to adulthood during upload (days 1–3/4) and the F_1_ generation to grow to mid-adulthood after living only in the spaceflight environment (days 3–8). We found that this duration exhausted our food source as evidenced by lack of visible food and presence of Dauer larvae. Reducing growth by 1 day resulted in viable worms of sufficient numbers for analysis (Table 2 and Figs. 2 and 6).

**Table 2.** Operation Scenarios Tested

Duration	Temperature profile	Outcome
8 Days	20°C	Not viable, ran out of food
7 Days	20°C	Viable, no Dauer larvae
1 Day	12°C	Viable
+6.5 Days	20°C	
8 Days	12°C	Some tests ran out of food around day 5.5 at 20°C
+6.5 Days	20°C	
3 Days	12°C	Viable
+6 Days	20°C	
8 Days	12°C	Viable, food close to exhaustion
+6 Days	20°C	
8 Days	NASA 12°C phase change	Ran out of food around day 5 at 20°C
+6 Days	20°C	
8 Days	NASA 10°C phase change	Viable
+6 Days	20°C	
5 Days	NASA 10°C phase change	Viable
+6 Days	20°C	

Unfortunately, with a maximum possible late turnover of payload at launch of—24 h, 3 days from turnover until installation in an incubator onboard the ISS was not a realistic timeline to produce viable animal cultures. Thus, for the timelines/late handover scenario associated with a Dragon launch scenario, similar to ICE-FIRST (Szewczyk *et al.*, 2008), we would require active temperature control throughout upload. We therefore moved on to test other scenarios that were, from an operations perspective, preferred.

We next tested the effect of cold incubation during the prelaunch (1 day) or prelaunch plus longest reported cold stowage upload via Dragon (Table 2, 8 days). For these tests, we also reduced the time of culturing at 20°C by half a day as we anticipated some level of development to occur while at 12°C. As shown in Table 2, chilling of the cultures for the day between handover and launch allowed for viable cultures, subjected to the same operational issues as when culturing at 20°C, whereas 8 days of chilling the cultures did not allow for all cultures tested to be viable.

We therefore tested short cold stowage upload (1 day handover to launch with 2 days from launch to the ISS) and longest reported cold stowage upload via Dragon (8 days) with a further half-day reduction in the time of culturing at 20°C. As shown in Table 2, both short and long cold stowage followed by 6 days of incubation produced viable cultures. These results suggested that even the longest duration cold stowage followed by a period of incubation onboard the ISS would be a suitable operational solution for MME.

As passive rather than active temperature control was preferred for upload operations, we tested the effect of passive cold stowage using NASA's phase change materials. Given that 12°C active temperature control during upload produced viable cultures, we first tested NASA's 12°C phase change materials temperature profile during long upload. As shown in Table 2, long cold stowage when using NASA's 12°C phase change materials temperature profile did not produce viable cultures, with worms running out of food about 1 day before the end of the incubation at 20°C.

This result may appear surprising; however, the phase change material starts at 10°C and ramps up to 14°C over the period of cold stowage. Thus, from an engineering perspective, the 12°C phase change materials do indeed maintain 12°C ± 2°C, but from a biological perspective, this is quite different from the 12°C ± 0.5°C achieved via active temperature control. Confirming that 10°C phase change materials also maintain within +/−2°C, we tested long cold stowage using NASA's 10°C phase change materials temperature profile, which did produce viable cultures (Table 2).

Having confirmed a viable passive stowage upload scenario for MME, we finally tested the impact of a short upload scenario with the 10°C phase change materials (1 day turnover to launch, 3 days to station, 1 day to put in incubator) and found that this also produced viable cultures (Table 2). Thus, through various iterations of testing that included what we might do to culture worms in our own laboratory, to implementing operational restrictions imposed by launch and upload to the ISS coupled with a better understanding of passive cold stowage performance, we were able to achieve a viable experimental protocol. Importantly, the data from this testing also inform of the ability of the experimental protocol to perform in other, off-nominal scenarios.

### 3.6. Validation of launch procedures, upload timings, and simulated incubation on the ISS

**FIG. 7.**
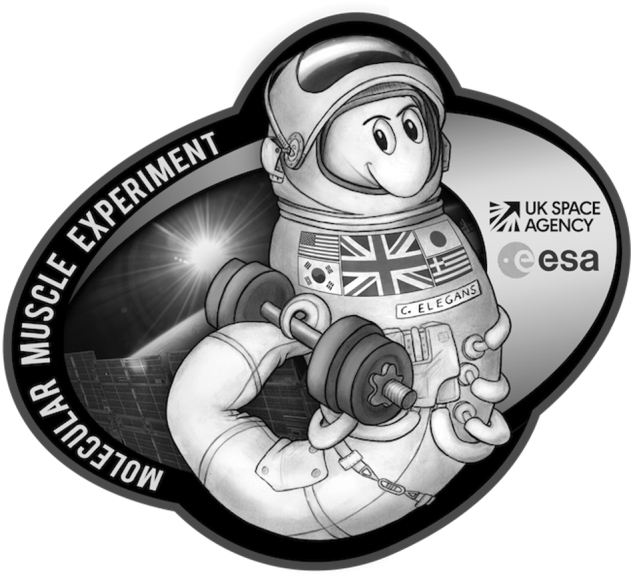
MME mission logo.

Having finalized an operationally acceptable upload scenario, we prepared for flight. First, to help increase the visibility of our outreach efforts (Gaffney *et al.*, 2018b), we developed and finalized a mission logo (Fig. 7). This was distributed to ∼2000 members of the public, mainly school children, between final ground testing and launch and created a “brand” for our experiment that was used by the UK government and media, which reached an estimated 41 million people.

**FIG. 8.**
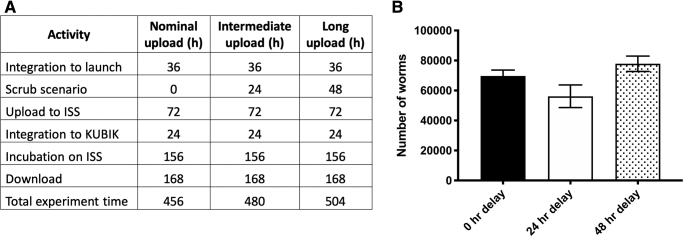
Final experimental timeline verification test. **(A)** Timing details for nominal upload, 24 h, and 48 h delays to launch. **(B)** Population growth after the full experiment following a nominal upload, and a simulated 24 and 48 h delay. One-way ANOVA is not significant between the three groups. ANOVA, analysis of variance.

Second, in consultation with ESA, we agreed a nominal launch scenario to be used in our final testing (Fig. 8A) as well as to assess the impact of a 24- and 48-h launch delay on our finalized experimental timeline. Note that a notional period of time (7 days) was used to simulate time in the Minus Eighty Laboratory Freezer for ISS (MELFI) before download of the samples back to Earth. As shown in Fig. 8B, cultures from the nominal and both launch delay scenarios grew as expected.

## 4. Discussion

The desired culture bag and EC hardware used in the past were not available for use with MME. Therefore, what should have been a straightforward repurposing of previously flown hardware became a program of work to redesign and revalidate flight hardware. This entailed dealing with the frequent challenges of successfully designing and flying an experiment to the ISS: use and validation of nonstandard laboratory hardware and validation of experimental timings for upload of biologically active experiments to the ISS.

We evaluated three culture bags based on past use in *C. elegans* flight experiments. Unsurprisingly, two of the bags selected to replicate bags used in previous flight performed well. However, one of the bags selected, made of FEP, was not compatible with worm growth despite having previously been used. This result was particularly striking as FEP is reported to have better gas exchange properties than PE and is why FEP bags are used to fly cell culture experiments (Vassy *et al.*, 2001). Upon closer inspection of the specifications of the PE and FEP bags, it was clear that the PE bags were half as thick as the FEP bags and therefore performed better as a gas exchange surface despite worse performance as a material.

As both PE and FEP bags are nonstandard equipment for growing *C. elegans*, these findings highlight the importance of properly testing proposed flight hardware before flight, as selecting FEP bags for better gas exchange and ease of use properties (*e.g.*, the capacity to load animals into bags via injection ports) was the operational preference before testing. Having uncovered that gas exchange did appear to be a concern for growing worms in plastic bags, we tested if culture volume to gas exchange surface area impacted culture growth. Unsurprisingly, we found that it did, which enabled us to optimize culture volume for MME, something not done for ICE-FIRST (Szewczyk *et al.*, 2008).

We also examined worm oxygen consumption using the diet from ICE-FIRST, CeMM (Szewczyk *et al.*, 2008), and the diet from CERISE, OP50 (Higashitani *et al.*, 2009). Consistent with the biological changes for worms grown in CeMM (Szewczyk *et al.*, 2003, 2006), we found that the worms consumed less oxygen. Collectively, our results suggest that growing worms in plastic culture bags does pose a challenge to worm experiment viability. The use of CeMM rather than OP50 as a food source may therefore reduce the oxygen exchange requirement and thus increase the viability of worm experiments conducted inside plastic culture bags on the ISS, but this comes with the added challenge that CeMM is more prone to become contaminated.

Regardless, it remains clear that proper ground testing is required preflight despite past successful *C. elegans* spaceflight experiments. Additionally, in contrast to challenges with experimental bags and gas exchange, the development of new ECs, similarly dependent on effective surface area to volume ratios, presented no problems. Gas exchange in the ECs was capable of producing a viable experiment. This result suggests that for future experiments it is likely that gas exchange between the *C. elegans* culture and ISS will be limited principally by the exchange properties of the culture bags and not the ECs.

The second most challenging portion of our preflight testing was testing and finalizing suitable scenarios for upload to the ISS that were consistent both with current operational realities and with successfully conducting our experiment. The data on durations from payload handover until integration on the ISS provided us with a realistic range of upload timings and should prove useful for others planning to upload via Dragon with NASA cold stowage.

Using these timings, we were able to establish that a *C. elegans* payload can be kept in a stasis during cold upload at 8–12°C, then subsequently grow and reproduce effectively. Of note, while the use of phase change material removed the need for active temperature control during upload, it was critical that we tested the performance of the phase change material temperature profile. Indeed, for biologically active cultures, phase change material provides a significantly different temperature profile than active temperature control.

## 5. Conclusion

In conclusion, we have updated the flight hardware and upload conditions available to support *C. elegans* experiments onboard the ISS. These validations provide a platform for MME and future investigators to successfully, and efficiently, conduct experiments onboard the ISS.

## Abbreviations Used

CeMM: C. elegans maintenance media
ECs: experiment cassettes
ESA: European Space Agency
FEP: fluorinated ethylene propylene
ISS: International Space Station
JAXA: Japanese Aerospace Exploration Agency
MELFI: Minus Eighty Laboratory Freezer for ISS
MME: Molecular Muscle Experiment
OCR: oxygen consumption rates
PE: polyethylene

## Notes

Associate Editor: Petra Rettberg

## References

[B1] Corsi A.K., Wightman B., and Chalfie M. (2015) A transparent window into biology: a primer on Caenorhabditis elegans. Genetics 200:387–407 26088431 10.1534/genetics.115.176099 PMC4492366

[B2] Demontis G.C., Germani M.M., Caiani E.G., Barravecchia I., Passino C., and Angeloni D. (2017) Human pathophysiological adaptations to the space environment. Front Physiol 8:547 28824446 10.3389/fphys.2017.00547 PMC5539130

[B3] Fitts R.H., Trappe S.W., Costill D.L., Gallagher P.M., Creer A.C., Colloton P.A., Peters J.R., Romatowski J.G., Bain J.L., and Riley D.A. (2010) Prolonged space flight-induced alterations in the structure and function of human skeletal muscle fibres. J Physiol 588:3567–3592 20660569 10.1113/jphysiol.2010.188508 PMC2988519

[B4] Gaffney C.J., Bass J.J., Barratt T.F., and Szewczyk N.J. (2014) Methods to assess subcellular compartments of muscle in *C. elegans*. J Vis Exp e52043. doi:10.3791/52043 PMC4354018 25489753

[B5] Gaffney C.J., Fomina E., Babich D., Kitov V., Uskov K., and Green D.A. (2017) The effect of long-term confinement and the efficacy of exercise countermeasures on muscle strength during a simulated mission to Mars: data from the Mars500 study. Sport Med Open 3:40 10.1186/s40798-017-0107-y PMC5684057 29134470

[B6] Gaffney C.J., Pollard A., Barratt T.F., Constantin-Teodosiu D., Greenhaff P.L., and Szewczyk N.J. (2018a) Greater loss of mitochondrial function with ageing is associated with earlier onset of sarcopenia in C. elegans. Aging (Albany. NY) 10:3382–3396 30455409 10.18632/aging.101654 PMC6286836

[B7] Gaffney C.J., Pollard A., Deane C., Cooke M., Balsamo M., Hewitt J., Vanapalli S., Szewczyk N., Etheridge T., and Phillips B. (2018b) Worms in space for outreach on earth: Space Life Science activities for the classroom. Gravit Space Res 6:74–82

[B8] Hewitt J.E., Pollard A.K., Lesanpezeshki L., Deane C.S., Gaffney C.J., Etheridge T., Szewczyk N.J., and Vanapalli S.A. (2018) Muscle strength deficiency and mitochondrial dysfunction in a muscular dystrophy model of Caenorhabditis elegans and its functional response to drugs. Dis Model Mech 11:dmm036137 30396907 10.1242/dmm.036137 PMC6307913

[B9] Higashibata A., Hashizume T., Nemoto K., Higashitani N., Etheridge T., Mori C., Harada S., Sugimoto T., Szewczyk N.J., Baba S.A., Mogami Y., Fukui K., and Higashitani A. (2016) Microgravity elicits reproducible alterations in cytoskeletal and metabolic gene and protein expression in space-flown Caenorhabditis elegans. NPJ Microgravity 2:15022 28725720 10.1038/npjmgrav.2015.22 PMC5515518

[B10] Higashitani A., Hashizume T., Sugimoto T., Mori C., Nemoto K., Etheridge T., Higashitani N., Takanami T., Suzuki H., Fukui K., Yamazaki T., Ishioka N., Szewczyk N., and Higashibata A. (2009) C. elegans RNAi space experiment (CERISE) in Japanese Experiment Module KIBO. Biol Sci Space 23:183–187 20729992 10.2187/bss.23.183 PMC2924584

[B11] Honda Y., Higashibata A., Matsunaga Y., Yonezawa Y., Kawano T., Higashitani A., Kuriyama K., Shimazu T., Tanaka M., Szewczyk N.J., Ishioka N., and Honda S. (2012) Genes down-regulated in spaceflight are involved in the control of longevity in Caenorhabditis elegans. Sci Rep 2:487 22768380 10.1038/srep00487 PMC3390002

[B12] Honda Y., Honda S., Narici M., and Szewczyk N.J. (2014) Spaceflight and ageing: reflecting on Caenorhabditis elegans in space. Gerontology 60:138–142 24217152 10.1159/000354772

[B13] Ishioka N., and Higashibata A. (2019) Space experiments using C. elegans as a model organism. Handbook of Space Pharmaceuticals, 1–32. doi:10.1007/978-3-319-50909-9_3-1

[B14] Kaletta T., and Hengartner M.O. (2006) Finding function in novel targets: C. elegans as a model organism. Nat Rev Drug Discov 5:387–399 16672925 10.1038/nrd2031

[B15] Koopman M., Michels H., Dancy B.M., Kamble R., Mouchiroud L., Auwerx J., Nollen E.A., and Houtkooper R.H. (2016) A screening-based platform for the assessment of cellular respiration in Caenorhabditis elegans. Nat Protoc 11:1798–1816 27583642 10.1038/nprot.2016.106 PMC5040492

[B16] Oczypok E.A., Etheridge T., Freeman J., Stodieck L., Johnsen R., Baillie D., and Szewczyk N.J. (2012) Remote automated multi-generational growth and observation of an animal in low Earth orbit. J R Soc Interface 9:596–599 22130552 10.1098/rsif.2011.0716 PMC3262433

[B17] Selch F., Higashibata A., Imamizo-Sato M., Higashitani A., Ishioka N., Szewczyk N.J., and Conley C.A. (2008) Genomic response of the nematode Caenorhabditis elegans to spaceflight. Adv Sp Res 41:807–815 10.1016/j.asr.2007.11.015 PMC2288577 18392117

[B18] Shen P., Yue Y., Zheng J., and Park Y. (2018) Caenorhabditis elegans: a convenient in vivo model for assessing the impact of food bioactive compounds on obesity, aging, and Alzheimer's disease. Annu Rev Food Sci Technol 9:1–22 29261338 10.1146/annurev-food-030117-012709

[B19] Simpson R.J., Kunz H., Agha N., and Graff R. (2015) Exercise and the regulation of immune functions. Prog Mol Biol Transl Sci 135:355–380 26477922 10.1016/bs.pmbts.2015.08.001

[B20] Strollo F., Gentile S., Strollo G., Mambro A., and Vernikos J. (2018) Recent progress in space physiology and aging. Front Physiol 9:1551 30483144 10.3389/fphys.2018.01551 PMC6240610

[B21] Szewczyk N.J., Kozak E., and Conley C.A. (2003) Chemically defined medium and Caenorhabditis elegans. BMC Biotechnol 3:19 14580264 10.1186/1472-6750-3-19 PMC270041

[B22] Szewczyk N.J., Mancinelli R.L., McLamb W., Reed D., Blumberg B.S., and Conley C.A. (2005) Caenorhabditis elegans survives atmospheric breakup of STS-107, Space Shuttle Columbia. Astrobiology 5:690–705 16379525 10.1089/ast.2005.5.690

[B23] Szewczyk N.J., Udranszky I.A., Kozak E., Sunga J., Kim S.K., Jacobson L.A., and Conley C.A. (2006) Delayed development and lifespan extension as features of metaboliclifestyle alteration in C. elegans under dietary restriction. J Exp Biol 209:4129–4139 17023606 10.1242/jeb.02492

[B24] Szewczyk N.J., Tillman J., Conley C.A., Granger L., Segalat L., Higashitani A., Honda S., Honda Y., Kagawa H., Adachi R., Higashibata A., Fujimoto N., Kuriyama K., Ishioka N., Fukui K., Baillie D., Rose A., Gasset G., Eche B., Chaput D., and Viso M. (2008) Description of international Caenorhabditis elegans experiment first flight (ICE-FIRST). Adv Sp Res 42:1072–1079 10.1016/j.asr.2008.03.017 PMC2493420 22146801

[B25] Trappe S., Costill D., Gallagher P., Creer A., Peters J.R., Evans H., Riley D.A., and Fitts R.H. (2009) Exercise in space: human skeletal muscle after 6 months aboard the International Space Station. J Appl Physiol 106:1159–1168 19150852 10.1152/japplphysiol.91578.2008

[B26] Vassy J., Portet S., Beil M., Millot G., Fauvel-Lafeve F., Karniguian A., Gasset G., Irinopoulou T., Calvo F., Rigaut J.P., and Schoevaert D. (2001) The effect of weightlessness on cytoskeleton architecture and proliferation of human breast cancer cell line MCF-7. FASEB J 15:1104–1106 11292682 10.1096/fj.00-0527fje

[B27] Warren P., Golden A., Hanover J., Love D., Shephard F., and Szewczyk N.J. (2013) Evaluation of the fluids mixing enclosure system for life science experiments during a commercial Caenorhabditis elegans spaceflight experiment. Adv Space Res 51:2241–2250 23794777 10.1016/j.asr.2013.02.002 PMC3684985

[B28] Zhang L., Gualberto D.G., Guo X., Correa P., Jee C., and Garcia L.R. (2015) TMC-1 attenuates C. elegans development and sexual behaviour in a chemically defined food environment. Nat Commun 6:6345 25695879 10.1038/ncomms7345

